# Explicit recognition of emotional facial expressions is shaped by expertise: evidence from professional actors

**DOI:** 10.3389/fpsyg.2013.00382

**Published:** 2013-06-28

**Authors:** Massimiliano Conson, Marta Ponari, Eva Monteforte, Giusy Ricciato, Marco Sarà, Dario Grossi, Luigi Trojano

**Affiliations:** ^1^Neuropsychology Laboratory, Department of Psychology, Second University of NaplesCaserta, Italy; ^2^Division of Psychology and Language Sciences, University College of LondonLondon, UK; ^3^Post Coma and Rehabilitation Care Unit, San Raffaele CassinoCassino, Italy

**Keywords:** facial expressions, conscious recognition, emotions, expertise, simulation, mentalizing

## Abstract

Can reading others' emotional states be shaped by expertise? We assessed processing of emotional facial expressions in professional actors trained either to voluntary activate mimicry to reproduce character's emotions (as foreseen by the “Mimic Method”), or to infer others' inner states from reading the emotional context (as foreseen by “Stanislavski Method”). In explicit recognition of facial expressions (Experiment 1), the two experimental groups differed from each other and from a control group with no acting experience: the Mimic group was more accurate, whereas the Stanislavski group was slower. Neither acting experience, instead, influenced implicit processing of emotional faces (Experiment 2). We argue that expertise can selectively influence explicit recognition of others' facial expressions, depending on the kind of “emotional expertise”.

## Introduction

Identification of emotional facial expressions is an ability of high surviving value that can be accomplished through activation of two main mechanisms, i.e., simulation and mentalizing (Goldman and Sripada, 2005; Decety and Grezès, 2006; Schulte-Rüther et al., 2007; Apperly, 2008; Bastiaansen et al., 2009). On one hand, an attributor can understand the mental state of an agent by covertly mimicking or reenacting the same activity of the agent without producing an overt behaviour (Goldman, 2002). An observer can attribute a mental state to a target by replicating the target's state in her/his own mind and assigning the output of this process to the target; the observer could also test a hypothesized state by mentally simulating it and verifying whether its outcome matches that of the target (Gallese and Goldman, 1998). On other hand, mentalizing, also known as theory of mind (Frith and Frith, 1999), can be conceived of as those higher cognitive operations devoted to infer other people's mental states from their actions, gestures, faces and the surrounding context. Such an information-based approach would play a crucial role in social interactions, because inferential processes would enable humans to decode other people's intentions and to modify behaviour accordingly (Blakemore, 2010).

A specifically regards recognition of other's emotions, it is well-known that observation of another person's emotional facial expression elicits a corresponding expression in the onlooker (Niedenthal, 2007). Several electromyographic studies have revealed covert facial muscle activity during observation of emotional faces; facial muscles involved in production of specific emotional expressions are also activated during the observation of the very same emotional faces (Dimberg and Thunberg, 1998; Dimberg et al., 2000). On the other hand, individuals can infer and attribute an emotion to a target by employing their knowledge on correspondence between particular facial configurations and emotional labels (Goldman and Sripada, 2005). Recent neurofunctional studies showed that mentalizing-related brain areas, such as medial frontal cortex or temporo-parietal junction, are activated during recognition of facial expressions (Schulte-Rüther et al., 2007; Peelen et al., 2010; Mattavelli et al., 2011).

Simulation and mentalizing can both operate in an explicit or an implicit way (Decety and Grezès, 2006; Keysers and Gazzola, 2006; Goldman, 2009). As regards simulation, recent data from developmental and adult neuropsychology showed that simulation of other's emotions can be accomplished by recruitment of implicit or explicit processes related to activation of involuntary or voluntary motor pathways (Oberman et al., 2009; Pistoia et al., 2010). With respect to mentalizing, it has been proposed a distinction between an earlier developing path, allowing implicit monitoring of other's mental states in a social environment, and a later developing path, more dependent on general cognitive functions that allows explicit inference of others' mental states (Apperly and Butterfill, 2009). No study has investigated yet whether and how expertise in simulation and mentalizing can shape explicit and implicit processing of others' emotional states in adult life. To tackle these issues, two groups of professional actors trained to different acting methods underwent behavioral tasks requiring explicit recognition (Experiment 1) or implicit processing (Experiment 2) of emotional facial expressions.

Professional actors were experts either in the “Stanislavski Method” or in the “Mimic Method”, that are two popular acting techniques. The Method originally developed by the Russian actor and theatre director Constantin Sergeyevich Stanislavski assumes that, in order to act as realistically as possible, the actor has to immerse into the character so deeply that she/he “becomes” the character; to this aim, the actor has to disassemble the descriptions of character from the text to capture deep psychological traits and has to resort to her/his own repertory of emotional memories and mental images to produce a reliable performance on stage (Benedetti, 1982). Instead, the “Mimic Method”, developed in Italy by the theatre director Orazio Costa, requires the actor to train her/his natural imitative skills in order to gain and enhance the capacity to capture relevant aspects of the character's personality and behaviour. The actor learns to act through imitation and action rehearsal analogously to the physical training of an athlete (Boggio, 2001).

Here, we could predict that in the “Mimic actors” the extensive training to exploit voluntary mimicry to simulate emotions could enhance explicit recognition of facial expressions. On the contrary, in the “Stanislavski actors” the long-lasting exercise to explicitly think about the contents of someone else's mind by means of abstract representations should slow down the capacity to consciously recognize emotions from faces. Such a pattern of results would be consistent with neuropsychological evidence on brain-damaged patients showing that a defect of simulative processes can account for impairments of identification of facial expressions, thus suggesting that emotional faces engage simulation more than mentalizing (Goldman and Sripada, 2005). Moreover, we predicted that actors' expertise in consciously reading others' emotional states could affect explicit but not implicit processing of facial expressions. This prediction would fit evidence reviewed above underlining a distinction between processing systems involved in explicit and implicit reading of others' intentions and emotions (Apperly and Butterfill, 2009; Oberman et al., 2009; Pistoia et al., 2010).

## Methods

### Participants

Thirty professional actors recruited in three main Italian acting schools volunteered to participate in the experiment. Fifteen actors experts in the “Mimic Method” (10 female; mean age = 35.2 years, *SD* = 5.6) and 15 actors experts in the “Stanislavski Method” (10 female; mean age = 31.9, *SD* = 7.1) had completed a 4-year acting training course 1–4 years before the present study was performed, and, from then on, they were pursuing a variety of careers in many different acting fields (e.g., movies, TV series or theatrical plays). Before starting the study, we ascertained that all the professional actors did not receive specific training or were familiar with facial expressions from the Facial Action Coding System (FACS, Ekman and Friesen, 1978). Thirty subjects without any previous acting experience, matching actors for age and sex (20 female; mean age = 33.7 years, *SD* = 4.9), were recruited as controls. All participants were white Caucasian, right-handed and had normal or corrected to normal vision. The study was conducted in accordance with the ethical standards of Helsinki Declaration; written informed consent was obtained from all the subjects.

### Stimuli and procedure

**Figure 1:**
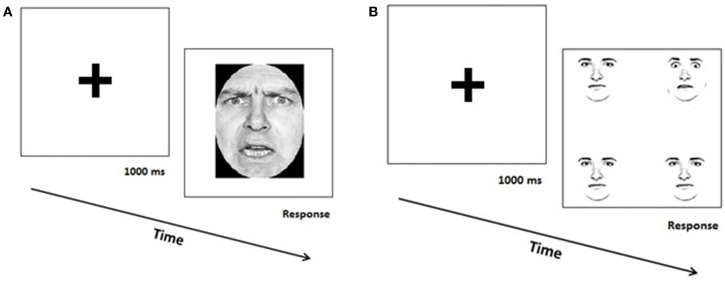
**Trial sequence in Experiment 1 showing an angry facial expression (A), and in Experiment 2 showing of a target-present condition with a target fearful expression (B)**.

In Experiment 1 (*explicit recognition of emotional facial expressions*) stimuli were photographs (8.6° × 10.4° of visual angle at a viewing distance of 60 cm) of 10 white Caucasian individuals (5 females) displaying a happy, fearful, angry, disgusted, surprised, or sad expression, selected from the “Pictures of Facial Affect” set (Ekman and Friesen, 1976); hair and non-facial areas were digitally occluded so that only the central face area was visible. The 60 experimental stimuli (10 items × 6 emotions) were preceded by six practice trials consisting in pictures of one additional model posing the six emotional expressions. For each stimulus, subjects were required to choose the expressed emotion among six labels (i.e., happiness, sadness, anger, fear, disgust and surprise; see Figure 1A). Participants provided their response by pressing with the right hand one of six keys of the QWERTY keyboard (“R, T, Y, U, I, O”) marked with the emotion labels, whose order was counterbalanced across subjects.

In Experiment 2 (*implicit processing of emotional facial expressions*) stimuli were obtained from photos of four white Caucasian individuals (two female and two male) displaying a happy, fearful, angry, disgusted, surprised, sad or emotionally neutral expression, selected from the Ekman and Friesen (1976) set; hair and non-facial areas were digitally occluded so that only the central face area was visible. In order to reduce perceptual variability, gray-scale images were converted to “line drawings” (17.6 pixels Gaussian Blur filter was applied). As a result, only pixels belonging to the eye, eyebrow, mouth, nose, and cheek regions were retained, while all other pixels were converted to white; after this conversion, one main difference among images was the emotional expression conveyed (Horstmann and Bauland, 2006; Krysko and Rutherford, 2009). Each stimulus included an array of 4 faces presented at the corners of an imaginary square, each face (4.6° × 5.8° of visual angle at a viewing distance of 60 cm) being equidistant from the centre. Half stimuli included one target face with an emotional expression and three neutral faces, with the target appearing randomly in one of the image locations (target-present). The other half stimuli included four faces with the same emotional expression (target-absent); each target-absent array was repeated 4 times. The complete stimulus set thus included 48 target-present items (2 identities × 6 emotional expressions × 4 spatial locations) and 48 target-absent items (2 identities × 6 emotional expressions × 4 repetitions), for a total of 96 trials; before starting the experiment 6 practice trials were given. Participants were instructed to concentrate on the fixation cross at the start of each trial, and then to determine whether there was a discrepant face or all faces were the same (see Figure 1B); it is worth underlining here that task instructions did not make reference to emotional information displayed by stimuli (Krysko and Rutherford, 2009). They had to respond by pressing one of two keys on the QWERTY keyboard (“B” or “H”), with stimulus-response mapping counterbalanced across subjects.

In both Experiments, stimuli were preceded by a fixation cross for 1000 ms and were centrally presented on a computer screen until subjects gave their response. Order of stimuli was randomized within Experiments; order of Experiments was counterbalanced across subjects. Participants were encouraged to respond as fast and correctly as possible; both Reaction Times (RTs, in milliseconds) and accuracy were recorded. Stimulus presentation and data collection were controlled by a PC using Cedrus SuperLab v.4.

At the end of the experiment, all participants underwent a semi-structured interview (debriefing session) in which they were asked several questions basically tapping four aspects: how they rated difficulty of either task, whether their own acting experience affected performance on either task, whether they feel that either task elicited emotional reactions, and whether the two tasks were solved in the same or different way.

## Results

### Experiment 1

A Two-Way mixed ANOVA, with emotion (disgust, happiness, fear, anger, surprise, and sadness) as a within-subject factor and group (Mimic group, Stanislavski group and controls) as a between-subject factor, was performed on accuracy and correct RTs.

**Figure 2:**
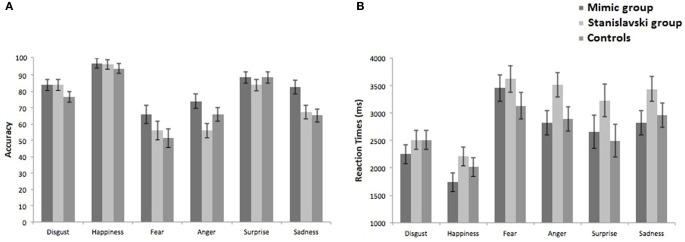
**Percentages of correct responses (A) and RTs (B) plotted against the six emotions, separately in the three groups.** Bars represents SEM.

Percentage of correct responses plotted against the six emotions are shown in Figure 2A separately for the three groups. The Two-Way mixed ANOVA revealed significant main effects of emotion, *F*_(5, 285)_ = 39.867, *p* = 0.0001; η^2^_p_ = 0.412, and group, *F*_(2, 57)_ = 13.621, *p* = 0.0001; η^2^_p_ = 0.323, whereas the emotion by group interaction was not statistically significant, *F*_(10, 285)_ = 1.547, *p* = 0.122, η^2^_p_ = 0.051. *Post-hoc* comparisons (paired t-tests) on the main effect of emotion showed that happiness, surprise, and disgust were significantly easier to be recognized than the other emotions (all *p* < 0.036, the significance threshold set according to False Discovery Rate, FDR, procedure for taking into account the number of multiple comparisons; Benjamini and Hochberg, 1995), whereas sadness, anger and fear did not differ between each other (*p* > 0.036). More relevantly, instead, *post-hoc* comparisons (unpaired *t*-tests, with the significance level set at *p* < 0.033 according to FDR procedure) on the main effect of group revealed that the Mimic group (82%) was significantly more accurate than both the Stanislavski (74%; *t* = −4.181, *p* = 0.0001) and the control group (73.7%; *t* = −4.868, *p* = 0.0001), whereas the Stanislavski and the control group did not differ between each other (−0.200, *p* = 0.842). These data strongly fitted with those previously reported in literature, and also patterns of confusions between emotion categories overlapped with available evidence (Ekman et al., 1987; Matsumoto, 1992; Jack et al., 2009). Actually, participants from the three groups mainly confused fear for surprise (from 23% to 29% on overall accuracy), disgust for anger (from 12% to 22%), anger for surprise (from 9% to 14%) and disgust (from 6% to 15%), surprise for fear (from 8% to 12%), and sadness for disgust (from 7% to 11%) and fear (from 7% to 11%).

Mean RTs plotted against the six emotions are shown in Figure 2B separately for the three groups. The Two-Way mixed ANOVA revealed significant main effects of emotion, *F*_(5, 285)_ = 24.067, *p* = 0.0001, η^2^_p_ = 0.297, and group, *F*_(2, 57)_ = 4.627, *p* = 0.014, η^2^_p_ = 0.140, whereas the emotion by group interaction was not statistically significant, *F*_(10, 285)_ = 0.991, *p* = 0.451, η^2^_p_ = 0.034. *Post-hoc* comparisons (paired t-tests) on the main effect of emotion generally confirmed RTs data: recognition of happiness and disgust was faster than recognition of fear, anger and sadness and recognition of surprise was faster than recognition of happiness and fear (but no comparison reached the significance threshold according to FDR procedure: *p* < 0.007). *Post-hoc* comparisons (unpaired *t*-tests) on the main effect of group revealed that the Stanislavski group was slower (3085 ms) than both the Mimic group (2624 ms, *t* = −2.271, *p* = 0.031) and controls (2667 ms, *t* = −2.357, *p* = 0.029); both comparisons were statistically significant according to FDR procedure for multiple comparisons (*p* < 0.016). The difference between the Mimic and the control group, instead, was far from the significance level (*t* = 0.328, *p* = 0.745).

### Experiment 2

Two-Way mixed ANOVAs, with emotion (disgust, happiness, fear, anger, surprise, and sadness) as a within-subject factor and group (Mimic group, Stanislavski group and controls) as a between-subject factor, was performed both on accuracy and correct RTs. Following previous studies (e.g., Krysko and Rutherford, 2009), the two ANOVAs were conducted separately for target-present and target-absent trials.

#### Target-present trials

**Figure 3:**
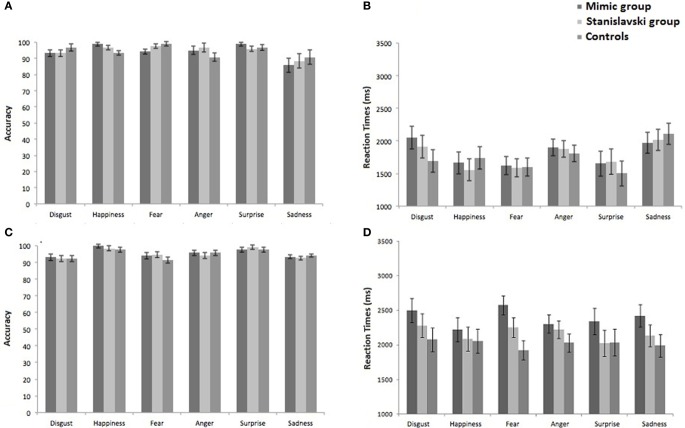
**Percentages of correct responses and RTs (bars represents SEM) plotted against the six emotions in the three groups, separately for target-present trials (A and B) and target-absent trials (C and D)**.

Percentage of correct responses plotted against the six emotions are shown in Figure 3A separately for the three groups. The Two-Way mixed ANOVA on accuracy revealed a significant main effect of emotion, *F*_(5, 210)_ = 6.19, *p* = 0.001; η^2^_p_ = 0.128, whereas the main effect of group, *F*_(2, 42)_ = 0.001, *p* = 0.991, η^2^_p_ = 0.000, and the emotion by group interaction, *F*_(10, 210)_ = 1.380, *p* = 0.189, η^2^_p_ = 0.062, were not statistically significant. *Post-hoc* comparisons (paired *t*-tests) on the main effect of emotion showed that recognition of sadness was significantly less accurate than happiness, fear, and surprise (all *p* < 0.001 with the significance level set at *p* < 0.033 according to FDR procedure). No significant differences were detected among all the other emotions (all *p* > 0.05).

Mean RTs plotted against the six emotions are shown in Figure 3B separately for the three groups. The Two-Way mixed ANOVA revealed a significant main effect of emotion, *F*_(5, 210)_ = 8.812, *p* = 0.0001; η^2^_p_ = 0.174, whereas the main effect of group, *F*_(2, 42)_ = 0.063, *p* = 0.939, η^2^_p_ = 0.003, and the emotion by group interaction, *F*_(10, 210)_ = 0.943, *p* = 0.494, η^2^_p_ = 0.043, were not statistically significant. *Post-hoc* comparisons (paired *t*-tests) performed on the main effect of emotion showed that recognition of sadness was significantly slower than recognition of happiness, fear and surprise (all *p* < 0.001 with the significance level set at *p* < 0.033 according to FDR procedure). No significant differences were detected among all other emotions (all *p* > 0.05).

#### Target-absent trials

Percentage of correct responses plotted against the six emotions separately in the three groups are shown in Figure 3C. The Two-Way mixed ANOVA did not reveal any significant main effect [emotion: *F*_(5, 210)_ = 1.32, *p* = 0.255, η^2^_p_ = 0.031; group: *F*_(2, 42)_ = 0.224, *p* = 0.799, η^2^_p_ = 0.011] or emotion by group interaction, *F*_(10, 210)_ = 0.590, *p* = 0.824, η^2^_p_ = 0.027.

Mean RTs plotted against the six emotions, separately in the three groups are shown in Figure 3D. The Two-Way mixed ANOVA did not reveal any significant main effect [emotion: *F*_(5, 210)_ = 0.940, *p* = 0.456, η^2^_p_ = 0.022; group: *F*_(2, 42)_ = 1.123, *p* = 0.245, η^2^_p_ = 0.044] or emotion by group interaction, *F*_(10, 210)_ = 0.671, *p* = 0.751, η^2^_p_ = 0.031.

### Debriefing session

Participants rated task difficulty in different way depending on the acting experience and on the kind of the experimental task. Actually, Stanislavski actors judged the explicit recognition task more difficult than the implicit processing task; Mimic actors, instead, did not appreciate substantial differences between the two tasks. When asked to report whether their performance might have been influenced by their acting technique, participants from both groups indicated that they thought their task performance was strongly influenced by the acting experience. As specifically regards Experiment 1, Stanislavski actors referred to be in discomfort during the task because they had to force themselves to analyse others' emotional states in an unusual way; actually, most of them (11/15) complained that facial cues are not sufficient to capture others' emotions. On the contrary, Mimic actors reported that the explicit recognition task put them in a rather “typical situation”, one which they are accustomed to in their acting experience. Participants from both groups generally stated that the explicit recognition task activated their own emotional reactions, whereas the implicit processing did not. In particular, most participants referred to have relied on visual analysis of perceptual differences among faces in each display, without paying relevant attention to the expressed emotion: eight Stanislavski actors and five Mimic actors reported to be completely unaware about the emotional nature of the task, and they did not realize that facial stimuli in the display conveyed emotional information.

## Discussion

The first main finding of the present study is the demonstration that recognition of emotional facial expressions can be shaped by experience in a highly specific fashion depending on the nature of the training experience. Actually, results of Experiment 1 showed that when required to explicitly identify emotions the Mimic group was significantly more accurate than both the Stanislavski group and controls, whereas the Stanislavski group was significantly slower than both the Mimic group and controls. By contrast, no effect of acting training was found on the performance on the implicit task for both groups (Experiment 2).

Classical studies on the effect of expertise on visual processing have emphasized the role of pure visual perceptual mechanisms in favoring effective post-training performance on visual discrimination tasks (Tanaka and Taylor, 1991; Schyns and Rodet, 1997; Tanaka et al., 2005). Recently, instead, a number of behavioral and neurofunctional studies have suggested that motor, action-related mechanisms can play a crucial role in visual expertise (Calvo-Merino et al., 2006; Casile and Giese, 2006; Cross et al., 2006, 2009; Aglioti et al., 2008).

In a seminal behavioral study, Casile and Giese (2006) showed that when blindfolded subjects learned to perform new complex action patterns by means of verbal and haptic feedback they also improved their ability to discriminate the same actions visually. This motor-mediated enhancement of action recognition is subserved by increased neural activity in premotor and parietal areas: expert male and female dancers show higher parieto-premotor activation while observing ballet moves from their own motor repertory compared to opposite-gender moves that they frequently see but do not perform (Calvo-Merino et al., 2006). Accordingly, learning complex dance patterns modulates neural motor activity during the observation of practiced as compared to visually familiar, but unpracticed, movements (Cross et al., 2006, 2009). The present results demonstrated that not only simulative processes involved in action recognition but also those related to more complex aspects of social cognition, such as emotion recognition, are affected by expertise. Following the idea that experts can “read the kinematics” of observed action they are trained to (Aglioti et al., 2008), here we suggest that the advantage of “Mimic actors” in the explicit emotion recognition was related to the voluntary engagement of their own facial musculature to understand others' emotional states. In such a task, mentalizing can even interfere with understanding of emotional states. Actually, slower performance of Stanislavski actors might be ascribed to their specific acting technique, which imposes them to deeply analyze not only other's mimicry but also the context in which the emotional state emerges. Therefore, when task requirements forced Stanislavski actors to extract emotional information exclusively from facial expression, their “mentalizing training” likely slowed down performance. Although at the moment this interpretation lacks of a direct manipulation check (external measures demonstrating different mentalizing and simulation abilities in the two groups), it seems to be consistent with a study on mind-reading skills of psychotherapists. Hassenstab et al. (2007) found that therapists did not differ from controls when making inferences based on facial cues, i.e., explicit recognition of facial expressions (Ekman and Friesen, 1976), and the eye test (Baron-Cohen et al., 2001), whereas they were significantly better when making inferences based on observation of naturalistic videos (i.e., to report feelings, thoughts, and intentions of an actor involved in a dinner party), likely due to their tendency to adopt cognitive-based strategies to comprehend others' emotional states. The present results are also in keeping with the demonstration that practice can modify emotional responses to observation of others' pain: while observing needles being inserted into others' body parts, physicians experts in acupuncture showed activation of areas involved in mentalizing and emotion regulation (e.g., medial prefrontal cortices and the temporo-parietal junction) and not in regions underpinning the affective aspects of pain processing (e.g., the anterior insula, somatosensory cortex and periaqueductal gray; Cheng et al., 2007).

The second relevant point of the present study was that acting expertise did not modulate implicit processing of facial expressions (Experiment 2). In healthy individuals, observation of happy and angry faces determines a corresponding mimic response in the observer (Dimberg and Thunberg, 1998; Dimberg et al., 2000). This facial response is spontaneous (i.e., without external prompting or a goal to mimic; Dimberg and Lundquist, 1988), unconscious (i.e., it occurs even when faces are presented subliminally), and rapid (i.e., it emerges within 1 s after face presentation; Dimberg et al., 2000). By contrast, voluntary facial expressions are effortful and slow (Dimberg et al., 2002), are affected by contextual demands and involve different neurofunctional mechanisms (Ekman, 1992; Morecraft et al., 2004). Recent neuropsychological data support that voluntary facial motility contributes to explicit but not implicit processing of emotional facial expressions. Actually, autistic individuals, whose spontaneous mimicry is impaired, perform tasks requiring explicit recognition of facial expressions as well as normal controls (Oberman et al., 2009). On the contrary, severely motor-disabled patients with complete paralysis of voluntary facial movements are selectively impaired in explicit recognition of facial expressions (Pistoia et al., 2010). On this basis, we suggest that extensive training in simulating emotions through conscious and voluntary activation of facial mimicry would guarantee a high level of accuracy in explicit, but not implicit, recognition of others' emotional states. By the same token, the “mentalizing training” of Stanislavski actors modulated performance on the explicit but not the implicit task, thus revealing that explicit and implicit mentalizing processes can be dissociated. Senju et al. (2009) demonstrated that individuals with Asperger syndrome adequately solve tasks requiring explicit mentalizing (verbal false-belief tasks), whereas they are impaired in measures of spontaneous, implicit mentalizing (i.e., anticipatory eye movements while viewing false-belief scenes). According to Senju et al. (2009) spared explicit mentalizing in individuals with Asperger syndrome might be accounted for by compensatory learning mechanisms fostered in the pathological brain. Our experimental data provided clear evidence that learning processes can also shape mind-reading in the healthy brain. Observations from the debriefing session supported this conclusion by suggesting that participants were aware of relationships between their acting experience and their performance on the explicit recognition task. As regards the implicit processing task, instead, participants of both groups reported that they performed the task mainly relying on perceptual visual analysis, without paying attention to the expressed emotion; some participants were even not aware that facial stimuli in the display conveyed emotional information. The lack of differences between the two groups in the implicit processing experiment might thus be ascribed to the fact that participants mainly relied on matching of visual features, with only marginal processing of emotional information. It should be taken into account also that the different perceptual features of stimuli used in Experiments 1 and 2 might have biased our results. For instance, the visual angle in Experiment 1 was nearly double that of Experiment 2 (see Smith and Schyns, 2009 for evidence on the effect of viewing distance on identification of facial expressions). On these bases, further investigation are warranted to better clarify whether acting experience influences implicit processing of emotional faces.

## Conclusions

In a seminal study Ekman et al. (1983) investigated emotion-specific activity of the autonomic nervous system in professional actors by requiring them to pose specific facial expressions or reliving past emotional experiences. Results showed differences in emotion-specific autonomic patterns between voluntary activation of emotion-related mimicry and reactivation of emotion-related representations. Here, we demonstrated that expertise can shape explicit recognition but not implicit processing of facial expressions. Moreover, since we found that expertise in the Stanislavski or the Mimic methods differently affected performance on the explicit task, we could suggest that recognition of facial expressions can be shaped by experience in a highly specific fashion depending on the nature of the training experience. It is worth noticing here that in our experiments we employed highly prototypical stimuli, as in most studies on recognition of emotional states (for a review see Goldman and Sripada, 2005). However, one should take into account that laboratory setting cannot fully represent processing of emotional facial expressions in real-life situations (Hess and Blairy, 2001; Bastiaansen et al., 2009). Future investigations should assess recognition of emotional faces by means of paradigms employing more ecologically valid facial expressions, such as stimuli from the Matsumoto and Ekman's (1988) Japanese and Caucasian Facial Expressions of Emotions (JACFEE).

### Conflict of interest statement

The authors declare that the research was conducted in the absence of any commercial or financial relationships that could be construed as a potential conflict of interest.
